# High‐fat diet induces protein kinase A and G‐protein receptor kinase phosphorylation of β_2_‐adrenergic receptor and impairs cardiac adrenergic reserve in animal hearts

**DOI:** 10.1113/JP273314

**Published:** 2017-02-02

**Authors:** Qin Fu, Yuting Hu, Qingtong Wang, Yongming Liu, Ning Li, Bing Xu, Sungjin Kim, Nipavan Chiamvimonvat, Yang K. Xiang

**Affiliations:** ^1^ Department of Pharmacology, School of Basic Medicine, Tongji Medical CollegeHuazhong University of Science and TechnologyWuhanChina; ^2^ The Key Laboratory for Drug Target Research and Pharmacodynamic Evaluation of Hubei ProvinceWuhanChina; ^3^ Department of PharmacologyUniversity of CaliforniaDavisCAUSA; ^4^ Institute of Clinical Pharmacology, Key Laboratory of Anti‐inflammatory and Immune Medicine, Ministry of Education, Collaborative Innovation Centre of Anti‐inflammatory and Immune MedicineAnhui Medical UniversityHefeiChina; ^5^ Shuguang HospitalShanghai University of Traditional Chinese MedicineShanghaiChina; ^6^ Division of Cardiovascular Medicine, Department of MedicineUniversity of CaliforniaDavisCAUSA; ^7^ VA Northern California Healthcare SystemMatherCAUSA

**Keywords:** ardiac function, beta adrenergic receptor, high fat diet

## Abstract

**Key points:**

- Patients with diabetes show a blunted cardiac inotropic response to β‐adrenergic stimulation despite normal cardiac contractile reserve.
- Acute insulin stimulation impairs β‐adrenergically induced contractile function in isolated cardiomyocytes and Langendorff‐perfused hearts.
- In this study, we aimed to examine the potential effects of hyperinsulinaemia associated with high‐fat diet (HFD) feeding on the cardiac β_2_‐adrenergic receptor signalling and the impacts on cardiac contractile function.
- We showed that 8 weeks of HFD feeding leads to reductions in cardiac functional reserve in response to β‐adrenergic stimulation without significant alteration of cardiac structure and function, which is associated with significant changes in β_2_‐adrenergic receptor phosphorylation at protein kinase A and G‐protein receptor kinase sites in the myocardium.
- The results suggest that clinical intervention might be applied to subjects in early diabetes without cardiac symptoms to prevent further cardiac complications.

**Abstract:**

Patients with diabetes display reduced exercise capability and impaired cardiac contractile reserve in response to adrenergic stimulation. We have recently uncovered an insulin receptor and adrenergic receptor signal network in the heart. The aim of this study was to understand the impacts of high‐fat diet (HFD) on the insulin–adrenergic receptor signal network in hearts. After 8 weeks of HFD feeding, mice exhibited diabetes, with elevated insulin and glucose concentrations associated with body weight gain. Mice fed an HFD had normal cardiac structure and function. However, the HFD‐fed mice displayed a significant elevation of phosphorylation of the β_2_‐adrenergic receptor (β_2_AR) at both the protein kinase A site serine 261/262 and the G‐protein‐coupled receptor kinase site serine 355/356 and impaired adrenergic reserve when compared with mice fed on normal chow. Isolated myocytes from HFD‐fed mice also displayed a reduced contractile response to adrenergic stimulation when compared with those of control mice fed normal chow. Genetic deletion of the β_2_AR led to a normalized adrenergic response and preserved cardiac contractile reserve in HFD‐fed mice. Together, these data indicate that HFD promotes phosphorylation of the β_2_AR, contributing to impairment of cardiac contractile reserve before cardiac structural and functional remodelling, suggesting that early intervention in the insulin–adrenergic signalling network might be effective in prevention of cardiac complications in diabetes.

## Key points

- Patients with diabetes show a blunted cardiac inotropic response to β‐adrenergic stimulation despite normal cardiac contractile reserve.
- Acute insulin stimulation impairs β‐adrenergically induced contractile function in isolated cardiomyocytes and Langendorff‐perfused hearts.
- In this study, we aimed to examine the potential effects of hyperinsulinaemia associated with high‐fat diet (HFD) feeding on the cardiac β_2_‐adrenergic receptor signalling and the impacts on cardiac contractile function.
- We showed that 8 weeks of HFD feeding leads to reductions in cardiac functional reserve in response to β‐adrenergic stimulation without significant alteration of cardiac structure and function, which is associated with significant changes in β_2_‐adrenergic receptor phosphorylation at protein kinase A and G‐protein receptor kinase sites in the myocardium.
- The results suggest that clinical intervention might be applied to subjects in early diabetes without cardiac symptoms to prevent further cardiac complications.

**Abbreviations**
AR: adrenergic receptor
AUC: area under the curve
β_2_AR: β_2_‐adrenergic receptor
EF: ejection fraction
FS: fractional shortening
G_i_: inhibitory guanine nucleotide‐binding protein
GRK: G‐protein receptor kinase
HFD: high‐fat diet
IR: insulin receptor
IRS: insulin receptor substrates
ISO: isoproterenol
IVRT: isovolumetric relaxation time
KO: knockout
maximal dP/dt: maximum values of the first derivative of left ventricular pressure
minimal dP/dt: minimal values of the first derivative of left ventricular pressure
NC: normal chow
PLB: phospholamban
PKA: protein kinase A
SERCA: sarco/endoplasmic reticulum Ca^2+^‐ATPase
TnI: troponin I
WT: wild‐type

## Introduction

Population‐based studies have shown that patients with type 2 diabetes have two to three times the risk of heart disease when compared with the general population (Bell, 2003; From *et al*. 2006). The increased incidence of heart failure in diabetic patients persists despite correction for age, hypertension, hypercholesterolaemia and coronary artery disease (Boudina & Abel, 2007). The pathogenesis of diabetic cardiomyopathy is multifactorial. Several hypotheses have been proposed, including metabolic derangements, abnormalities in ion homeostasis, alteration in structural proteins and interstitial fibrosis (Boudina & Abel, 2007). One of the hallmarks in type 2 diabetes is insulin resistance and the associated increase in the concentrations of circulating insulin, free fatty acids and glucose, which are risk factors for diabetic cardiomyopathy (Fang *et al*. 2004). Many studies have documented the effects of fatty acids and glucose in the myocardium, including dysregulation of metabolism and autophagy and an increase in oxidative stress (Ansley & Wang, 2013; Kubli & Gustafsson, 2014). These alterations not only contribute to cardiac contractile dysfunction, but also promote cardiac remodelling and increase the risk of cardiac ischaemic events (Ansley & Wang, 2013; Mei *et al*. 2015). In contrast, little is known about the impacts of circulating insulin on diabetic hearts.

The prominent defect in diabetic hearts is diastolic dysfunction, with preservation of the ejection fraction (EF; Desai & Fang, 2008). A large number of diabetic patients also show a decrease in exercise tolerance before the onset of overt cardiac dysfunction (Scognamiglio *et al*. 1995), which has been attributed to autonomic neuropathy (Vinik & Ziegler, 2007). Others have reported that exercise‐intolerant patients also show a blunted inotropic response to dobutamine stimulation, suggesting that the cardiac β‐adrenergic system is affected (Scognamiglio *et al*. 1995). In agreement, cardiac preparations from streptozotocin‐induced diabetic rats display reduced inotropic and chronotropic responses to β‐adrenergic stimulation (Vadlamudi & McNeill, 1984; Yu & McNeill, 1991). These studies suggest that exercise intolerance and a blunted inotropic response to adrenergic stimulation can be considered as early signs of diabetic cardiomyopathy, preceeding clinical symptoms of cardiac complications. Mechanistically, in streptozotocin‐induced diabetic rats, the expression of cardiac β_1_‐adrenergic receptors (β_1_ARs) is significantly decreased, which may account for the depressed contractile response to adrenergic stimuli (Heyliger *et al*. 1982). Meanwhile, in leptin receptor‐deficient *db*/*db* mice, an impaired cardiac functional reserve capacity during maximal β‐adrenergic stimulation is linked with unfavourable changes in cardiac energy metabolism (Daniels *et al*. 2010). Therefore, the mechanisms involved in the early stages of myocardial adaptations, such as exercise intolerance and a blunted inotropic response to adrenergic stimulation, prior to an overt decline in mechanical performance, are still incompletely understood.

β‐Adrenergic signalling is essential in the regulation of cardiac function in response to stress. Both β_1_ARs and β_2_‐adrenergic receptors (β_2_ARs) are functionally coupled to the adenylate cyclase system, contributing to the cardiac response to β‐adrenergic stimulation (Dincer *et al*. 2001). In congestive and ischaemic human heart failure, elevated sympathetic drive can promote compensatory cardiac contractility. But chronic sympathetic stress leads to desensitization of βARs via several molecular mechanisms, including a decrease in the expression of β_1_ARs and in coupling of β_1_ARs to adenylate cyclase, and an increase in the expression of inhibitory G‐protein (G_i_) and G‐protein receptor kinases (GRKs; Liggett, 2001). The cardiac β_2_AR signalling is traditionally considered beneficial to the myocardium relative to the cardiac toxicity induced by β_1_ARs (Bernstein *et al*. 2005; Grundy, 2015). Phosphorylated β_2_ARs couple to G_i_ and promote Akt signalling (Wang *et al*. 2008), which protects myocytes against apoptosis induced by oxidative stress or chronic sympathetic β_1_AR signalling (Zhu *et al*. 2001). However, the same β_2_AR–G_i_ coupling can lead to inhibition of adenylate cyclase, which compromises cAMP and protein kinase A (PKA) activity and contributes to cardiac dysfunction in pressure‐overload transaortic constriction models (Du *et al*. 2000; Zhu *et al*. 2012). Taken together, these previous findings suggest that changes in β‐adrenergic signalling in the heart may play either a detrimental or a protective role depending on the pathological setting.

We have recently characterized a complex consisting of the insulin receptor (IR) and the β_2_AR in the heart (Fu *et al*. 2014). Furthermore, acute insulin stimulation promotes cross‐talk with the β_2_AR signalling pathway in a Gβγ‐independent, insulin receptor substrate‐dependent G‐protein receptor kinase (GRK)2 phosphorylation of β_2_ARs. Consequently, insulin impairs β‐adrenergically induced contractile function in isolated cardiomyocytes and in Langendorff‐perfused hearts (Fu *et al*. 2014). In the present study, we aimed to examine the potential effects of hyperinsulinaemia associated with feeding a high‐fat diet (HFD) on the cardiac IR–β_2_AR signalling network and the impacts on cardiac adaptation during early stages of diabetes. Mice lacking the β_2_AR (β_2_AR‐KO) were used to define whether the β_2_AR is a contributor to hyperinsulinaemia‐induced βAR signalling dysfunction. We analysed cardiac function at the baseline and after β‐adrenergic stimulation by i.p. injection of isoproterenol after 8 weeks of HFD feeding. Cardiac structural remodelling was analysed using immunohistochemical and biochemical analyses. Our results show that 8 weeks of HFD feeding leads to decreases in cardiac functional reserve in response to β‐adrenergic stimulation without significant alteration of cardiac structure and basal function. This decrease in cardiac functional reserve is associated with significant increases in the levels of phosphorylation of the β_2_AR at PKA and GRK sites in the heart.

## Methods

### Ethical approval

The animal care and experimental protocols conform to the principles of UK regulations (Grundy, 2015) and were approved by the institutional animal care and use committee of Tongji Medical College, Huazhong University of Science and Technology and the University of California at Davis (approval numbers 17407 and 19147).

### Experimental animals and *in vivo* treatment

C57BL/6 mice were purchased from Charles River and Beijing HFK Bioscience Co. Ltd, China. In brief, 50 wild‐type (WT) and 50 β_2_AR global knockout (β_2_AR‐KO) 5‐ to 6‐week‐old male C57BL/6J mice were randomly assigned to low‐fat or high‐fat diet for 8 weeks (*n* = 25 each group). The diets used for these studies were from Research Diets (New Brunswick, NJ, USA), with 10% kcal fat (catalogue no. D12450J) used as the control normal chow (NC) diet and 60% kcal fat diet (catalogue no. D12492) used as the HFD. The mice had *ad libitum* access to food and they were housed in a room with a 12 h light–12 h dark cycle.

### Intraperitoneal glucose tolerance test

Mice were fasted for 16 h and then challenged with glucose (1 g kg^−1^, i.p.). Blood samples were drawn from the tail vein immediately before the glucose challenge and at 30, 60, 90 and 120 min thereafter. Blood glucose concentrations were determined using a glucometer (ACCU‐CHEK, Roche, Mannheim, Germany). The area under the curve (AUC) was calculated to evaluate glucose tolerance.

### Insulin tolerance test and blood insulin detection

After a 6 h fast, mice were injected with insulin (0.75 U kg^−1^, i.p.; Sigma, St Louis, MO, USA). The blood glucose value and AUC were assessed before and at 30, 60, 90 and 120 min after injection of insulin. Blood serum was collected before glucose injection to measure the insulin concentration. Insulin was measured using ALPCO mouse ultrasensitive insulin ELISA kits (Salem, NH, USA) according to the manufacturer's protocol.

### Echocardiography

Echocardiography was performed using a Vevo 2100 imaging system from Visual Sonics (Toronto, ON, Canada) with a 22–55 MHz MS550D transducer. Mice were anaesthetized with isofluorane supplemented with 100% O_2_, and the body temperature, respiratory rate and ECG were constantly monitored. To minimize variation of the data, the heart rate was maintained at 400–450 beats min^−1^ during the measurements of cardiac function. Cardiac function was recorded at the baseline and after administration of the βAR agonist isoproterenol (ISO, 0.2 mg kg^−1^, i.p.; Sigma). Systolic function parameters, including the EF and fractional shortening (FS), were measured by two‐dimensional parasternal short‐axis imaging plane of M‐mode traces close to the papillary muscle level. Tissue Doppler imaging mode was applied to measure diastolic function as previously described (Qi *et al*. 2013; Zhang *et al*. 2013).

### Haemodynamic study

Haemodynamic measurement was carried out with a pressure catheter (SPR‐839; Millar Instruments, Houston, TX, USA) connected to an AD Instruments Power‐Lab 4/30 with Lab Chart Pro 7.0 software (MPVS‐300; AD Instruments, Colorado Springs, CO, USA). In brief, mice were anaesthetized by i.p. injection of ketamine and xylazine (80 and 5 mg kg^−1^, respectively, Sigma, St Louis, MO, USA) and placed in the supine position on a heat pad. A ventral mid‐line neck incision was made, the carotid artery separated from the vagus nerve and a pressure–volume catheter inserted via the carotid artery tip into the left ventricle. After 5–10 min of stabilization, the baseline pressure was recorded. To measure the β‐adrenergic response, 0.2 mg kg^−1^ ISO was injected i.p. Measurements and analysis were performed with Lab Chart Pro 7.0.

### Adult myocyte‐shortening assay

Adult mouse cardiomyocytes were isolated from WT and β_2_‐KO mice as indicated and cultured as described previously (Soto *et al*. 2009). Cells were placed in the middle of a glass‐bottomed dish with beating buffer (mm: NaCl, 120; KCl, 5.4; NaH_2_PO_4_, 1.2; MgSO_4_, 1.2; Hepes, 20; glucose, 5.5; and CaCl_2_, 1; pH 7.1) and allowed to settle for 10 min. Platinum electrodes were placed near the cells, and the cells were paced at 1 Hz with a voltage of 30 V using an SD9 stimulator (Grass Technology, Warwick, RI, USA) as described by Fu *et al*. (2015). The contractile shortening events of myocytes were recorded on an inverted microscope (Zeiss AX10; Dublin, CA, USA) at ×20 magnification, which allowed observation of eight to 10 rod‐shaped cardiomyocytes with clear striations per field of view using the MetaMorph program (Molecular Devices, Sunnyvale, CA, USA). The maximal shortening was analysed by MetaMorph and normalized to the baseline.

### Western blot analysis

Adult cardiomyocytes isolated from NC and HFD mice were stimulated with ISO (100 nmol l^−1^) after incubation with or without pertussis toxin (1 mg ml^−1^ for 3 h). Left ventricular extracts or adult cardiomyocyte lysates were homogenized at 4°C for Western blotting using standard methods previously described (Fu *et al*. 2014). Membranes were probed with antibodies directed at β_1_AR (V‐19, 1:500 dilution), β_2_AR (M‐20, 1:500 dilution), GRK2 (C‐15, 1:1000 dilution) and Gα_i_ (C‐10, 1:1000 dilution) from SCBT (Santa Cruz, CA, USA), phospho‐troponin I (Ser23/24, #4004, 1:2000 dilution) and troponin I (#4002, 1:2000 dilution) from Cell Signaling (Danvers, MA, USA), SERCA (2A7‐A1, 1:1000 dilution; Thermo, Rockford, IL, USA), phospho‐phospholamban (Ser16, 1:5000 dilution; Bradilla, London, UK), phospholamban (MA3‐922, 1:1000 dilution; Affinity Bioreagent, Golden, CO, USA) and γ‐tubulin (T6557, 1:5000 dilution; Sigma‐Aldrich, St Louis, MO, USA). The primary antibodies were revealed with fluorescent‐conjugated secondary antibodies using an Odyssey scanner (Li‐COR Biosciences, Lincoln, NE, USA). The signal was quantified by Image Studio software version 2.1 (Li‐COR Biosciences).

### Histology

Mouse hearts were perfused with 4% paraformaldehyde and fixed in paraformaldehyde for 24 h. Fixed hearts were paraffin embedded and serially sectioned at 5 μm on a microtome. Tissue sections were stained with Haematoxylin and Eosin to examine heart morphology. Masson's trichrome staining was used to examine myocardial fibrosis. The blue‐stained areas in Masson's trichrome staining and cardiomyocyte cross‐section in Haematoxylin and Eosin staining were measured using the ImageJ program (National Institutes of Health, Bethesda, MD, USA) by a researcher blinded to the samples. The quantification was performed in five regions of each heart.

### Quantitative real‐time PCR analysis

Total RNA was isolated from snap‐frozen hearts using RNAiso Plus (TaKaRa Bio Inc., Kusatsu, Shiga, Japan) according to the manufacturer's protocol. First‐strand cDNA was synthesized from RNA using a SMARTer PCR cDNA Synthesis Kit (TaKaRa Bio Inc.). Real‐time quantitative RT‐PCR was performed with a Step One Plus Real‐Time PCR system thermocycler (Thermo Fisher Scientific, Waltham, MA, USA) using SYBR Premix Ex Taq (Tli RNaseH Plus; TaKaRa Bio Inc.). All the primer sequences are listed in Table 1. The relative expression level of specific mRNA was determined by the comparative cycle threshold (C_T_) method ($2^{-\Delta \Delta C_{T}}$) normalized to endogenous control *GAPDH* gene.

**Table 1:** Quantitative RT/PCR primer sequences

Gene	Primer	Sequence
α‐Myosin heavy chain (α‐MHC)	Forward	5′‐CAACGCCAAGTGTTCCTC‐3′
	Reverse	5′‐AGCTCTGACTGCGACTCCTC‐3′
β‐Myosin heavy chain (β‐MHC)	Forward	5′‐ATGTGCCGGACCTTGGAAG‐3′
	Reverse	5′‐CCTCGGGTTAGCTGAGAGATCA‐3′
Atrial natriuretic peptide (ANP)	Forward	5′‐TCGTCTTGGCCTTTTGGCT‐3′
	Reverse	5′‐TCCAGGTGGTCTAGCAGGTTCT‐3′
Brain natriuretic peptide (BNP)	Forward	5′‐CTCCTGAAGGTGCTGTCC‐3′
	Reverse	5′‐GCCATTTCCTCCGACTTT‐3′
α‐Smooth muscle actin (αSMA)	Forward	5′‐GTCCCAGACATCAGGGAGTAA‐3′
	Reverse	5′‐TCGGATACTTCAGCGTCAGGA‐3′
Connective tissue growth factor (CTGF)	Forward	5′‐CACAGAGTGGAGCGCCTGTTC‐3′
	Reverse	5′‐GATGCACTTTTTGCCCTTCTTAATG‐3′
Glyceraldehyde 3‐phosphate dehydrogenase (GAPDH)	Forward	5′‐CATGGCCTTCCGTGTTCCTA‐3′
	Reverse	5′‐CCTGCTTCACCACCTTCTTGAT‐3′

### Statistical analysis

All statistical analyses were performed using GraphPad Prism 6 software (GraphPad Software Inc., La Jolla, CA, USA). Statistical analyses were performed using Student's two‐tailed unpaired *t* test, one‐way ANOVA followed by Tukey's *post hoc* test or two‐way repeated‐measures ANOVA with Bonferroni multiple comparisons test. A value of *P* < 0.05 was considered to indicate statistical significance. All data are expressed as means ± SEM. The sample size for each group is shown in the figure legends or tables.

## Results

**Figure 1:**
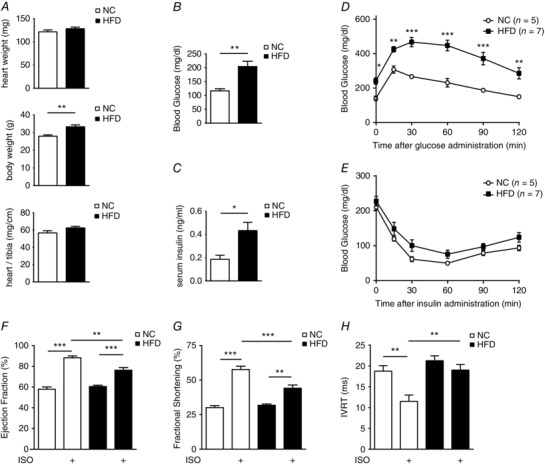
Feeding high‐fat diet (HFD) for 8 weeks induces impairment of cardiac functional reserve in response to adrenergic stimulation *A–C* show body weight (*A*), fasting blood glucose (*B*) and fasting serum insulin concentrations (*C*) after 8 weeks of normal chow (NC) or high‐fat diet (HFD) feeding. ^*^ *P* < 0.05 and ^**^ *P *< 0.01 between HFD and NC groups (*n* = 10). The HFD induces glucose intolerance (*D*) and mild insulin intolerance (*E*) when compared with NC control animals. ^*^ *P *< 0.05 and ^**^ *P* < 0.01 by two‐way repeated‐measures ANOVA with Bonferroni multiple comparisons test (*n* = 5–7). *F–H*, cardiac contractile function of the mice before and after β‐adrenergic stimulation [isoproterenol (ISO), 0.2 mg kg^−1^, i.p.] was measured by echocardiography after 8 weeks of HFD. Data show ejection fraction (*F*) and fractional shortening (*G*) measured by M‐mode, and isovolumetric relaxation time (IVRT; *H*) measured by tissue Doppler image mode. ^**^ *P *< 0.01 and ^***^ *P* < 0.001 (*n* = 8) by one‐way ANOVA followed by Tukey's *post hoc* test.

**Figure 2:**
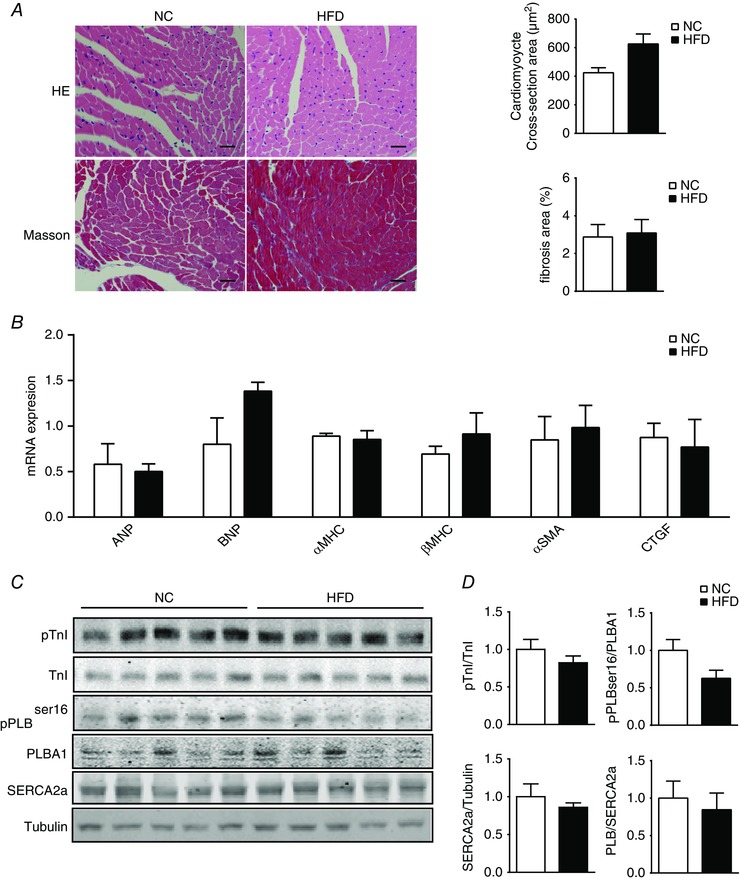
Mice after 8 weeks of HFD feeding display normal cardiac gene expression and morphology *A*, heart sections from NC and HFD mice were stained with Haematoxylin and Eosin (HE; scale bar = 100 μm) to examine heart morphology and stained with Masson's trichrome (scale bar = 100 μm) to examine fibrosis. The cardiomyocyte cross‐sectional areas and fibrosis‐positive areas were quantified and plotted (*n* = 3). *B*, RT‐PCR showing left ventricular expression of genes involved in cardiac hypertrophy and fibrosis in mice after 8 weeks of HFD. *C* and *D*, Western blots showing protein kinase A (PKA) phosphorylation of phospholamban (PLB) and troponin I (TnI), and SERCA2a expression in NC and HFD mouse hearts. [Color figure can be viewed at wileyonlinelibrary.com]

We fed C57BL/6J wild‐type mice with 60% HFD for 8 weeks. The HFD‐fed mice exhibited significant body weight gain compared with the mice fed with NC, which was associated with elevated fasting blood glucose and insulin (Fig. 1 *A*–*C*). The HFD mice also exhibited significant glucose intolerance and mild insulin intolerance relative to NC control animals (Fig. 1 *D* and *E*). The overall features indicated a stage of prediabetes or an early stage of diabetes associated with obesity. At this stage, echocardiographic analysis revealed that cardiac contractile function, including the systolic indices of EF and FS and the diastolic parameter isovolumetric relaxation time (IVRT), was normal in HFD animals relative to NC control animals (Fig. 1 *F*–*H* and Table 2). Consistent with the observed cardiac function, HFD hearts were grossly normal in morphology, with mild hypertrophy (Fig. 2 *A* and *B*). We also examined the cardiac contractile reserve in response to β‐adrenergic stimulation, which is usually impaired in patients with diabetes and/or metabolic syndrome (Scognamiglio *et al*. 1998). Despite the grossly normal cardiac structure and function, the cardiac contractile reserve in response to the β‐adrenergic agonist isoproterenol was impaired in HFD animals relative to NC control mice (Fig. 1 *F* and *G* and Table 2). Further examination showed that there was no significant change in expression of regulatory proteins in excitation–contraction coupling, including troponin I (TnI), phospholamban (PLB) and sarco/endoplasmic reticulum calcium ATPase 2 (SERCA2a; Fig. 2 *C* and *D*). Moreover, the basal levels of phosphorylation of PLB and TnI by PKA, which is essential for excitation–contraction coupling, were not altered in HFD hearts relative to NC control hearts (Fig. 2 *C* and *D*). Together, these observations suggest that 8 weeks of HFD feeding induced mild or early diabetes in mice, which was associated with impaired contractile reserve to adrenergic stress despite overall normal cardiac structure and function.

**Table 2:** Echocardiographic characteristics of NC and HFD mice treated with β‐adrenergic stimulation (ISO, 0.2 mg kg^−1^, i.p.)

Paramter	NC	NC + ISO	HFD	HFD + ISO
HR (beats min^−1^)	419 ± 14.42	603 ± 9.16***	411 ± 21.40	617 ± 13.10***
IVS;d (mm)	0.67 ± 0.05	0.83 ± 0.09	0.69 ± 0.06	0.81 ± 0.06
IVS;s (mm)	0.94 ± 0.10	1.39 ± 0.12*	1.28 ± 0.36	1.27 ± 0.12
LVID;d (mm)	3.67 ± 0.07	3.18 ± 0.08	3.64 ± 0.19	3.23 ± 0.26
LVID;s (mm)	2.53 ± 0.09	1.41 ± 0.10*	2.32 ± 0.26	1.69 ± 0.29
LVPW;d (mm)	0.73 ± 0.06	0.72 ± 0.07	0.79 ± 0.03	0.82 ± 0.11
LVPW;s (mm)	1.05 ± 0.06	1.17 ± 0.11	1.09 ± 0.07	1.2 ± 0.15
EF (%)	57.94 ± 2.22	88.25 ± 1.79***	60.54 ± 1.27	76.25 ± 2.58***##
FS (%)	30.07 ± 1.53	57.66 ± 2.45***	31.81 ± 0.92	44.14 ± 6.15***##
LV mass (mg)	88.04 ± 6.70	73.8 ± 7.06	94.91 ± 4.59	80.76 ± 6.67
LV mass (corrected)	68.84 ± 5.36	59.04 ± 5.65	75.93 ± 3.67	64.61 ± 5.33
LV Vol;d (μI)	58.43 ± 2.12	38.4 ± 3.82***	62.2 ± 3.46	40.91 ± 3.64***
LV Vol;s (μI)	24.66 ± 1.80	4.38 ± 0.75***	24.44 ± 1.22	9.9 ± 1.73***

Results are expressed as means ± SEM (*n* = 8 per group). ^*^ *P* < 0.05, ^**^ *P* < 0.01 and ^***^ *P* < 0.001 compared with the control group. ^##^ *P* < 0.01 compared with the NC + ISO group. Abbreviations: EF, ejection fraction; FS, fractional shortening; HFD, high‐fat diet; HR, heart rate; ISO, isoproterenol; IVS;s and IVS;d, interventricular septal wall thickness at systole and diastole; LVID;s and LVID;d, left ventricular dimension at systole and diastole; LV mass, left ventricular mass; LV mass (corrected), LV mass corrected for body surface area LVPW;s and LVPW;d, posterior wall thickness at systole and diastole; LV Vol;s and LV Vol;d, left ventricle volume at systole and diastole; and NC, normal chow.

**Figure 3:**
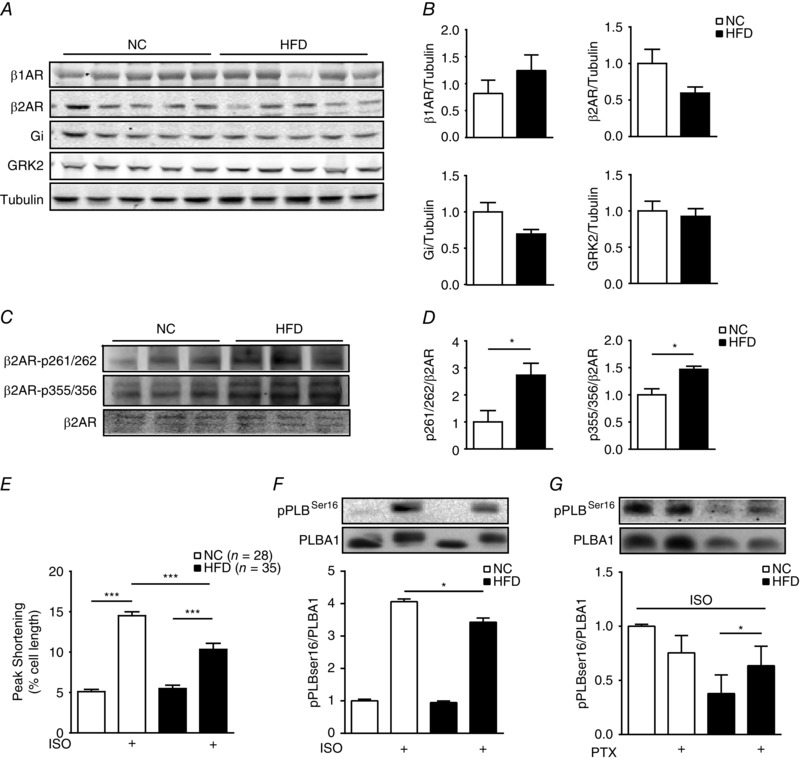
Feeding the HFD for 8 weeks induces an impaired contractility response to adrenergic stimulation in cardiomyocytes *A* and *B*, Western blots show the expression of cardiac β_1_‐adrenergic receptor (β_1_AR), β_2_‐adrenergic receptor (β_2_AR), G‐protein and G‐protein receptor kinase (GRK)2 in NC and HFD hearts. *C* and *D*, Western blots show the phosphorylation of β_2_AR at both PKA and GRK sites in NC and HFD hearts. ^*^ *P* < 0.05 by Student's *t* test between paired groups. *E*, data show contractile function in response to β‐adrenergic stimulation (ISO, 100 nm) in adult cardiac myocytes isolated from NC and HFD mice. ^***^ *P* < 0.001 by one‐way ANOVA followed by post hoc Tukey's test. *F* and *G*, data show PKA phosphorylation of PLB in response to β adrenergic stimulation (ISO, 100 nm) in the absence or presence of pertussis toxin (PTX, 1 μg ml^−1^, 3 h) in adult cardiomyocytes isolated from NC and HFD mice (*n* = 3). ^*^ *P* < 0.05 by Student's *t* test between paired groups.

We then set out to examine potential alterations in cardiac adrenergic signalling pathways in the HFD mice. The HFD hearts displayed normal expression of β_1_AR, β_2_AR and G‐protein. G‐Protein receptor kinase 2, a kinase that regulates cardiac adrenergic signalling and is usually upregulated in heart failure, was not altered either (Fig. 3 *A* and *B*). However, the β_2_ARs in HFD hearts displayed increased levels of phosphorylation at both the PKA site serine 261/262 and the GRK site serine 355/356 relative to NC control hearts (Fig. 3 *C* and *D*). To assess adrenergic signalling directly in cardiomyocytes, we isolated adult myocytes from NC and HFD mice to perform a contractile shortening assay. Myocytes from both NC and HFD mice exhibited similar contractile shortening at baseline (Fig. 3 *E*). However, myocytes from HFD mice exhibited a reduced contractile response to isoproterenol stimulation relative to NC control myocytes (Fig. 3 *E*). Consistently, myocytes from HFD mice exhibited a reduced PKA phosphorylation of PLB at serine 16 in response to isoproterenol stimulation relative to NC control myocytes (Fig. 3 *F*). We have recently shown that acute insulin stimulation promotes β_2_AR coupling to G_i_ in cardiomyocytes (Fu *et al*. 2014). Inhibition of G_i_ with pertussis toxin increased the isoproterenol‐induced PKA phosphorylation of PLB in cardiomyocytes from HFD mice (Fig. 3 *G*), indicating that isoproterenol triggered βAR activation of G_i_ in HFD cardiomyocytes. As a control, pertussis toxin did not affect isoproterenol‐induced phosphorylation of PLB in NC cardiomyocytes. Together, these data suggested that HFD feeding promoted desensitization of cardiac βAR via phosphorylation at both PKA and GRK sites, which resulted in a reduced contractile shortening response to adrenergic stress in the cardiomyocytes. Consequently, the reduced myocyte shortening is likely to contribute to the impaired cardiac contractile reserve in response to β‐adrenergic stimulation *in vivo* (Fig. 1).

**Figure 4:**
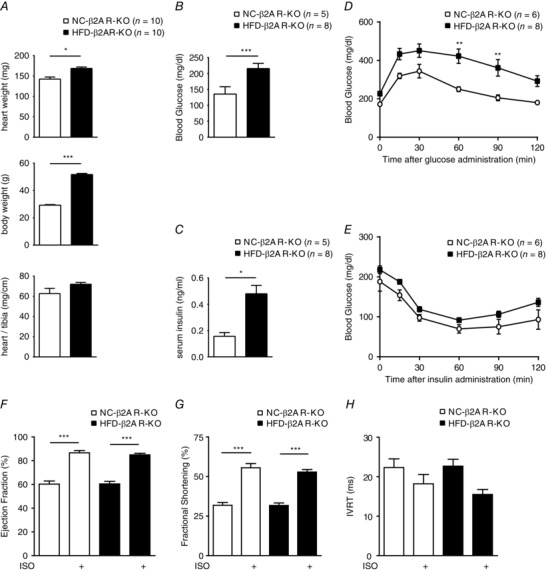
Deletion of the β_2_AR gene normalizes cardiac contractile response to adrenergic stimulation in HFD mice *A*–*C* show body weight (*A*), fasting glucose (*B*) and insulin concentrations (*C*) in mice lacking the β_2_AR (β_2_AR‐KO) after 8 weeks of NC or HFD feeding. ^*^ *P* < 0.05 and ^***^ *P* < 0.001 for HFD *vs* NC. Eight weeks of HFD feeding induces glucose intolerance (*D*) and mild insulin intolerance (*E*) when compared with NC control animals. ^*^P < 0.05 and ^**^P < 0.01 between groups by two‐way repeated‐measures ANOVA with Bonferroni multiple comparisons test. *F*–*H*, after 8 weeks of HFD feeding, cardiac contractile function in response to β‐adrenergic stimulation (ISO, 0.2 mg kg^−1^, i.p.) was examined by echocardiography in the β_2_AR‐KO mice. Data show ejection fraction (*F*) and fractional shortening (*G*) measured by M‐mode and isovolumetric relaxation time (*H*) measured by tissue Doppler image mode (*n* = 8). ^***^ *P* < 0.001 by one‐way ANOVA followed by Tukey's *post hoc* test.

**Figure 5:**
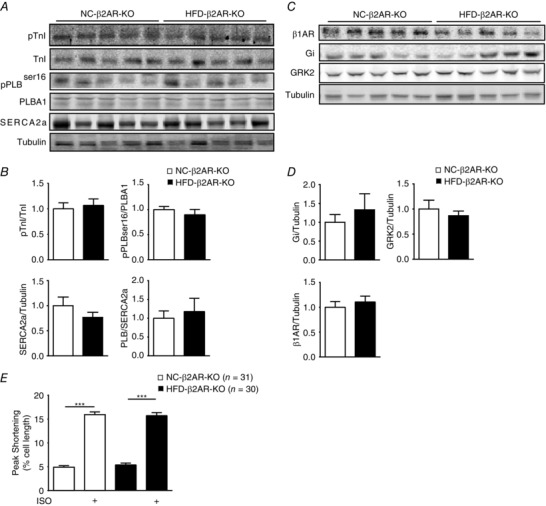
Deletion of the β_2_AR gene normalizes myocyte contractile response to adrenergic stimulation after HFD feeding Western blots show PKA phosphorylation of PLB and TnI, and SERCA2a expression (*A* and *B*), as well as expression of cardiac adrenergic signalling regulatory proteins in the hearts of β_2_AR‐KO mice fed NC and HFD (*C* and *D*). *E*, myocyte contractile shortening in response to β‐adrenergic stimulation (ISO, 100 nm) was examined in NC and HFD β_2_AR‐KO cardiomyocytes. ^***^ *P* < 0.001 by one‐way ANOVA followed by Tukey's *post hoc* test.

We then used mice lacking the β_2_AR gene to assess the role of the β_2_AR in HFD‐induced impairment of contractile reserve in hearts. After HFD feeding, β_2_AR‐KO mice exhibited significant body weight gain compared with the β_2_AR‐KO NC control mice, which was associated with elevated resting blood glucose and insulin, similar to those in WT mice (Fig. 4 *A*–*C*). The HFD‐fed β_2_AR‐KO mice also exhibited significant glucose intolerance and mild insulin intolerance relative to NC control animals (Fig. 4 *D* and *E*). Overall, echocardiographic analysis revealed that cardiac function was normal in HFD β_2_AR‐KO mice, including the systolic indices of EF and FS and the diastolic parameter IVRT (Fig. 4 *F*–*H*). In contrast to WT mice, mice lacking the β_2_AR exhibited normal cardiac contractile reserve in response to β‐adrenergic stimulation after HFD feeding (Fig. 4 *F* and *G*). There was no significant change in expression of TnI, PLB and SERCA2a pump in HFD β_2_AR‐KO hearts compared with NC β_2_AR‐KO mice (Fig. 5 *A* and *B*). The phosphorylation of TnI and PLB was also preserved in HFD‐fed β_2_AR‐KO hearts (Fig. 5 *A* and *B*). Moreover, HFD β_2_AR‐KO hearts exhibited normal expression of β_1_AR, G‐protein and GRK2 compared with NC β_2_AR‐KO mice (Fig. 5 *C* and *D*). Consistently, myocytes isolated from HFD β_2_AR‐KO mice exhibited normal contractile shortening in the resting state and after stimulation with isoproterenol (Fig. 5 *E*). Together, these data indicated that deletion of β_2_AR gene ameliorated HFD‐induced impairment of contractile reserve in hearts.

**Figure 6:**
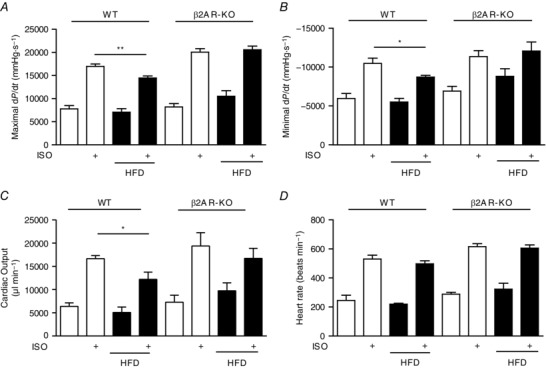
Deletion of the β_2_AR gene normalizes the cardiac functional reserve response to adrenergic stimulation after HFD feeding After HFD feeding, cardiac haemodynamics were measured in wild‐type (WT) and β_2_AR‐KO mice before and after i.p. injection of ISO (0.2 mg kg^−1^). The maximal d*P*/d*t* (*A*), minimal −d*P*/d*t* (*B*), cardiac output (*C*) and heart rate (*D*) were recorded and plotted in bar graphs (*n* = 6). ^*^ *P* < 0.05 and ^**^ *P* < 0.01, by Student's unpaired *t* test between groups.

We also assessed cardiac function directly with haemodynamic studies *in vivo*. In WT mice, 2 months of HFD feeding did not affect contractile properties at the baseline, including maximal d*P*/d*t* (maximum values of the first derivative of left ventricular pressure 7789 ± 699.9 *vs*. 7078 ± 748.5 mmHg s^−1^), minimal d*P*/d*t* (minimum values of the first derivative of left ventricular pressure −5962 ± 640.3 *vs*. −5502 ± 461.5 mmHg s^−1^) and cardiac output (6380 ± 758.3 *vs*. 5078 ± 1164 μl min^−1^), but significantly impaired cardiac contractility in response to administration of isoproterenol, including maximal d*P*/d*t* (16,973 ± 506.9 *vs*. 14,472 ± 432.8 mmHg s^−1^), minimal d*P*/d*t* (−10,466 ± 673.6 *vs*. −8710 ± 673.6 mmHg s^−1^) and cardiac output (16,648 ± 668.2 *vs*. 12,203 ± 1548 μl min^−1^; Fig. 6 *A*–*D*). In comparison, deletion of β_2_AR normalized cardiac contractility in HFD mice, including maximal d*P*/d*t* (8196 ± 724.3 *vs*. 10,504 ± 1212 mmHg s^−1^), minimal d*P*/d*t* (−6910 ± 613.4 *vs*. −8808 ± 978.5 mmHg s^−1^) and cardiac output (7274 ± 1502 *vs*. 9733 ± 1721 μl min^−1^), and rescued cardiac contractile reserve in response to administration of isoproterenol, including maximal d*P*/d*t* (20,046 ± 746.7 *vs*. 20,576 ± 792.8 mmHg s^−1^), minimal d*P*/d*t* (−11,339 ± 769.9 *vs* −12,053 ± 1170 mmHg s^−1^) and cardiac output (19,388 ± 2871 *vs*. 16,719 ± 2161 μl min^−1^; Fig. 6 *A*–*D*).

## Discussion

β‐Adrenergic receptors are the key regulators of cardiac function in response to stress. In heart diseases, βARs are often desensitized via phosphorylation as a result of elevated sympathetic drive (Brum *et al*. 2006). Here, we show that 2 months of HFD feeding promotes phosphorylation of cardiac β_2_ARs at both PKA and GRK sites, attenuation of myocyte contractile shortening *in vitro* and impairment of cardiac contractile reserve *in vivo* in response to adrenergic stimulation. The HFD‐induced attenuation of the adrenergic response occurs without significant alteration of the cardiac structure and basal function. These data provide direct evidence that 2 months of HFD feeding promotes phosphorylation of β_2_ARs, which is correlated with the compromised cardiac stress response that is present in HFD‐fed mice before structural remodelling occurs in hearts.

In a classic view, during augmented circulatory demands in clinical conditions such as diabetes mellitus, the increased sympathetic activity and subsequent βAR stimulation is an important physiological mechanism to enhance cardiac performance. However, the chronic increase of cardiac adrenergic drive could in turn promote β_1_AR desensitization, internalization and/or downregulation (Brodde *et al*. 2006), contributing to the development and progression of diabetic cardiomyopathy (Aneja *et al*. 2008; Tillquist & Maddox, 2012) and heart failure (Lymperopoulos *et al*. 2013; Florea & Cohn, 2014). In comparison, β_2_ARs are known to couple functionally to both stimulatory G protein (G_s_) and G_i_ to modulate cAMP production (Grundy, 2015), and the phosphorylation of β_2_AR promotes a coupling switch from G_s_ to G_i_ (Daaka *et al*. 1997), which induces Akt signalling to protect myocytes against apoptosis (Zhu *et al*. 2001). However, the same β_2_AR–G_i_ coupling can lead to inhibition of cAMP production by adenylate cyclase and subsequent PKA activity, which has been shown to contribute to cardiac dysfunction in pressure‐overload transaortic constriction models (Du *et al*. 2000; Zhu *et al*. 2012). In the present study, HFD feeding promoted phosphorylation of cardiac β_2_ARs at PKA and GRK sites, decreased PKA phosphorylation of PLB and attenuated myocyte shortening and cardiac contractile reserve in response to β‐adrenergic stimulation. Incubation with the G_i_ inhibitor pertussis toxin increased isoproterenol‐induced phosphorylation of PLB in isolated HFD cardiomyocytes, confirming that isoproterenol triggered receptor activation of G_i_ to shunt the β_2_AR signal away from PKA activation. Deletion of the β_2_AR restored the contractile response in cardiomyocytes isolated from HFD mice and normalized contractile reserve in response to adrenergic stress. Together, these data indicate that HFD feeding promoted both phosphorylation of β_2_ARs and the receptor coupling to inhibitory G‐protein to diminish contractile reserve in the mice.

β‐Adrenergic receptors are known to undergo heterologous desensitization mediated by secondary messenger‐dependent kinases, including PKA and protein kinase C (Chuang *et al*. 1996; Gainetdinov *et al*. 2004). Recent studies show that a growing list of neurohormonal factors and cytokines can promote desensitization of cardiac βARs and contribute to cardiac dysfunction via divergent cross‐talk mechanisms *in vivo* (Vasudevan *et al*. 2013; Tilley *et al*. 2014). For example, tumour necrosis factor α promotes GRK‐dependent phosphorylation of β_2_ARs, which attenuates adrenergic signalling and exacerbates cardiac dysfunction in a pressure‐overload model with transaortic constriction (Vasudevan *et al*. 2013). Block of tumour necrosis factor α signalling ameliorates cardiac dysfunction in mice. Interestingly, tumour necrosis factor α induces GRK2‐mediated desensitization of βARs in a Gβγ‐independent fashion. Coincidentally, insulin also promotes a Gβγ‐independent GRK2 phosphorylation of β_2_AR in the heart, which is dependent on IR and insulin receptor substrates (Daaka *et al*. 1997; Fu *et al*. 2014). These observations suggest that GRK2 functions as a key factor to promote desensitization of cardiac βARs independent of sympathetic drive. As a result, tumour necrosis factor α and insulin are linked to cardiac adrenergic regulation by promoting βAR phosphorylation and/or desensitization, which can either directly induce cardiac dysfunction or exacerbate cardiac pathophysiology in heart failure. Notably, recent clinical trials of diabetic medications (e.g. glyburide) in heart failure patients show adverse effects (Basu *et al*. 2007; Pantalone *et al*. 2012), suggesting that aggressive metabolic controls with insulin secretagogues might exacerbate cardiac symptoms. This may be attributable to these medications driving IR‐mediated phosphorylation of β_2_AR. On the other hand, a hyperinsulinaemia‐driven phosphorylation of β_2_ARs might offer an explanation for the lack of benefit from β‐blocker therapy in heart failure patients with diabetes mellitus (Haas *et al*. 2003). Together, these studies suggest the therapeutic potential of targeting hyperinsulinaemia and the Gβγ‐independent GRK2 signalling pathway in heart failure associated with diabetes.

In summary, we show that cardiac β‐adrenergic signalling is impaired in the early stages of diabetes after 8 weeks of HFD feeding, which occurs before structural change in the heart. This study also suggests that clinical intervention might be applied to subjects with early diabetes without cardiac symptoms to prevent further cardiac complications.

## Additional information

### Competing interests

None declared.

### Author contributions

Q.F, Y.K.X. and N.C. conceived and designed the experiments. Q.F., Y.T.H., Q.W., Y.L., B.X., N.L. and S.K. collected, assembled, analysed and interpreted the data. Q.F. and Y.K.X. wrote the manuscript. All authors have approved the final version of the manuscript and agree to be accountable for all aspects of the work in ensuring that questions related to the accuracy or integrity of any part of the work are appropriately investigated and resolved. All persons designated as authors qualify for authorship, and all those who qualify for authorship are listed.

### Funding

This study was supported by China National Natural Science Foundation of China grants 81102438 and 81473212 (Q.F.) and 81428022 (Y.K.X.) and by National Insitute of Health grants HL127764 and HL112413 (Y.K.X.) and S10 OD10389 (Y.K.X.). Y.K.X. is an established investigator of the American Heart Association and a Shanghai Eastern Scholar.
